# Simvastatin-Loaded Chitosan-Functionalized PLGA Nanoparticles: Characterization and Use in Intimal Hyperplasia Therapy

**DOI:** 10.3390/pharmaceutics17030391

**Published:** 2025-03-20

**Authors:** Ashley A. Peters, Chanpreet Kaur, Maleen Cabe, Kelly A. Langert, Kristopher Maier, Vivian Gahtan

**Affiliations:** 1 Department of Surgery, Loyola University of Chicago, Maywood, IL 60153, USA; apeters19@luc.edu (A.A.P.);; 2 Research Service, Edward Hines Jr., VA Hospital, Hines, IL 60141, USA; 3 Department of Molecular Pharmacology and Neuroscience, Loyola University of Chicago, Maywood, IL 60153, USA; 4 Surgical Service, Edward Hines Jr., VA Hospital, Hines, IL 60141, USA

**Keywords:** chitosan, poly-lactic-co-glycolic acid, nanoparticles, intimal hyperplasia, peripheral arterial disease, statins

## Abstract

**Background**: Statins have beneficial pleiotropic effects, including reducing intimal hyperplasia (IH), but off-target effects remain a concern. Here, we tested the hypothesis that chitosan-functionalized polymeric nanoparticles (NPs) loaded with simvastatin (SL-cNPs) would (1) readily associate with endothelial cells (ECs) and vascular smooth muscle cells (VSMCs); (2) affect EC and VSMC function; and (3) reduce IH compared to systemic simvastatin. **Methods**: Human aortic ECs and VSMCs were cultured with fluorescently labeled SL-cNPs. The association of SL-cNPs was assessed by immunostaining and flow cytometry. The effect of SL-cNPs, empty cNPs (E-cNPs), and free simvastatin on cells was determined using qRT-PCR for RhoA and RhoB. Carotid artery balloon-injured rats were treated intraoperatively with intraluminal saline, E-cNPs, low- or high-dose SL-cNPs, periadventitial high-dose SL-cNPs, or with pre- and post-operative oral simvastatin plus intraoperative intraluminal saline or low-dose SL-cNPs. Rats were euthanized (day 14) and IH was quantified. **Results**: SL-cNPs readily associated with ECs and VSMCs. Low- and high-dose SL-cNPs induced significant increases in EC and VSMC RhoA gene expression. High-dose SL-cNPs induced a significant increase in EC RhoB expression, while free simvastatin and low- and high-dose SL-cNPs significantly increased RhoB expression in VSMCs. In vivo, oral simvastatin plus intraluminal SL-cNPs significantly reduced IH compared to controls. **Conclusions**: cNPs can be used as a vehicle to locally deliver statins to vascular cells. However, other NP formulations may be preferential for IH reduction given only the combination of oral simvastatin and SL-cNPs effectively reduced IH.

## 1. Introduction

Restenosis secondary to intimal hyperplasia (IH) after percutaneous transluminal balloon angioplasty (PTA) for peripheral arterial disease (PAD) remains an ongoing challenge that leads to reintervention and poor patient outcomes [1,2,3,4]. Depending on the location and type of intervention, secondary occlusion ranges from 20% to 60% [5]. To help mitigate this important clinical problem, understanding the pathophysiology of IH is crucial. IH is triggered by endothelial cell (EC) denudation and vascular smooth muscle cell (VSMC) injury [3,6]. The endothelial damage then stimulates the production of proinflammatory molecules and activation of circulatory monocytes that initiate excessive neointima formation through VSMC migration, VSMC proliferation, and excessive extracellular matrix deposition [3,6,7,8]. While the application of drug eluting systems, such as paclitaxel-coated balloons and stents, was a major advance in the prevention of IH for coronary artery occlusive disease, this technology remains controversial in PAD management [4,9,10,11,12]. Therefore, the lack of effective treatments for the prevention of IH in PAD after PTA represents an important gap in our knowledge.

**Figure 1:**
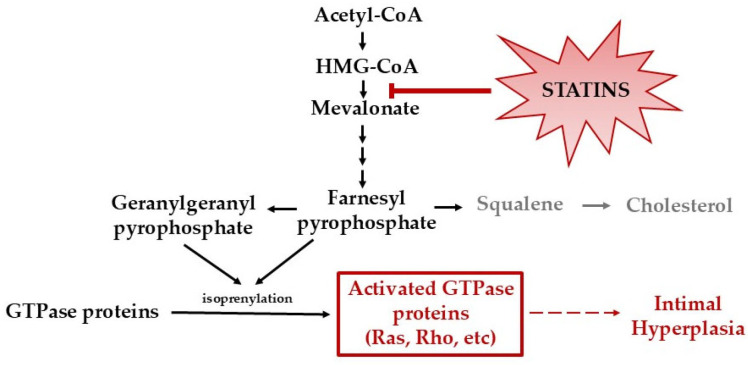
Mevalonate/cholesterol synthesis pathway. Statins inhibit HMG-CoA reductase, leading to inhibition of cholesterol synthesis and isoprenoid intermediates. The inhibition of isoprenoid intermediates disrupts the activation of G proteins, including Ras and Rho.

Statins are competitive inhibitors of 3-hydroxy-3-methyl-glutaryl-CoA (HMG-CoA) reductase, blocking a rate-limiting step in the mevalonate/cholesterol synthesis pathway [13]. In addition to reducing cholesterol levels, inhibition of HMG-CoA also prevents the production of isoprenoid intermediates. This in turn disrupts the signaling of small G proteins, including Ras and Rho, that are post-translationally modified by isoprenoids. The inhibition of isoprenoid intermediates and disruption of G protein signaling largely enable statins’ known beneficial pleiotropic effects, including IH reduction (Figure 1) [14,15,16]. For example, statins have been demonstrated to (1) reduce VSMC migration and proliferation [17]; (2) attenuate vascular inflammation by reducing leukocyte adhesion and trans-endothelial migration [18,19,20,21,22]; and (3) accelerate reendothelialization by mobilizing, differentiating, and improving the survival of resident and circulating endothelial progenitor cells [23]. In animal models, oral simvastatin has been shown to reduce IH by 25% [24]. Unfortunately, in the clinical realm, statins are not tolerated in nine percent of patients due to negative off-target effects, such as muscle-related symptoms (myalgia, myositis, rhabdomyolysis), cognitive decline, hepatoxicity, new onset diabetes, and peripheral neuropathy [17]. Furthermore, systemic delivery of statins, such as oral administration, has not proven to be highly effective in preventing IH due to statins’ low solubility, rapid metabolism, and low bioavailability [25]. Because of these issues, research has focused on improving in vivo drug delivery through the use of localized therapies. Currently, to maximize statins’ pleiotropic effects while minimizing systemic toxicity, studies are investigating the use of statins loaded into drug delivery carriers, including nanoparticles (NPs) [25,26]. While NPs have demonstrated the ability to increase the solubility, stability, and absorption of statins, no FDA-approved statin-loaded NP for clinical use currently exists [25].

Poly-lactic-co-glycolic acid (PLGA) is an FDA-approved biodegradable polymer that can be formed into NPs that passively target tissues and release incorporated agents in a controlled, localized fashion [27]. Recent preclinical studies have investigated polymeric NPs in cancer and cardiovascular research, with one study investigating pitavastatin eluting stents in coronary artery disease [25,28,29]. While this study demonstrated promise towards IH treatment with nanotechnology, variations in polymeric NP coating now raise questions concerning the optimal delivery vehicle(s) for IH and its effect on VSMC and EC physiology. Chitosan, a naturally occurring biopolymer derived from crustacean shells, is polycationic. Studies have indicated coating NPs with positively charged chitosan improves adhesion and solubility compared to PLGA alone, thereby optimizing the release of the embedded agents [30,31].

The purpose of this study was to determine the feasibility and therapeutic efficacy of administering localized, intraluminal or periadventitial simvastatin-loaded chitosan-PLGA NPs (SL-cNPs) on IH in a rat carotid artery injury model. Given that many PAD interventions require balloon angioplasty without stent placement, this study focuses on an intraluminal or periadventitial therapy that would not require the placement of a stent. We hypothesized that SL-cNPs would (1) readily associate with ECs and VSMCs in vitro; (2) affect EC and VSMC gene expression; and (3) reduce IH compared to systemic simvastatin in vivo.

## 2. Materials and Methods

### 2.1. Materials

Human aortic ECs, human VSMCs, and corresponding cell culture media were purchased from Cell Applications, Inc. (San Diego, CA, USA). RNA extraction was performed utilizing QIAGEN RNA extraction kits (Germantown, MD, USA). All PCR primers were from Applied Biosystems (Waltham, MA, USA).

For nanoparticle synthesis, ester-terminated poly(lactic-co-glycolic) acid (PLGA 85:15) was obtained from LACTEL (Birmingham, AL, USA). Dichloromethane (DCM), acetonitrile (MeCN), dimethyl sulfoxide (DMSO), poly(vinyl alcohol) (PVA, 31,000–50,000 Da, 87–89% hydrolyzed), and chitosan (low molecular weight (50,000–190,000 Da, 75–85% deacetylated) were purchased from Sigma-Aldrich (St. Louis, MO, USA). Simvastatin was obtained from Cayman Chemical (Ann Arbor, MI, USA). 1,1′-dioactadecyl-3,3,3′,3′-tetramethylindodicarbocyanine, 4-chlorobenzenesulfonate salt (DiD) was from Thermofisher Scientific (Waltham, MA, USA).

Simvastatin for rat chocolate-flavored treats was purchased from Fisher Scientific (Hampton, NH, USA) and sent to Bio-Serv (Flemington, NJ, USA) where the rodent-specific oral simvastatin chocolate-flavored treats were manufactured (4 mg simvastatin/treat, to correlate to a simvastatin dosing of 10 mg/kg). Pluronic^TM^ F-127 gel (20% solution in DMSO) for periadventitial placement of SL-cNPs was purchased from Thermo Fisher Scientific (Eugene, OR, USA).

### 2.2. Nanoparticle Synthesis

The detailed characterization of chitosan-functionalized PLGA nanoparticles (cNPs) is described in further detail in previously published work [31]. Briefly, PLGA nanoparticles (NPs) and cNPs were prepared by oil-in-water single emulsion, using commercially available polymer (85:15, viscosity 0.55–0.75). Chitosan stock solution was made by dissolving chitosan powder in 1% acetic acid to form 1% (*w*/*v*) and diluted 1:1 with PVA to make solutions. PLGA polymer (100 mg) was first dissolved in an organic solvent (0.6 mL MeCN and 0.4 mL DCM) and then added dropwise into an aqueous solution containing 5% PVA with or without 0.5% chitosan (6 mL) under vigorous vortexing. The emulsion was formed by sonication (sonication amplitude 70%) using an ultrasonic processor (GE130PB, Cole-Parmer, Vernon Hills, IL, USA) for 10 rounds of 30 s on and 30 s off. After sonication, the emulsified mixture was added to a 1 L beaker containing PVA solution (0.5% PVA with or without 0.5% chitosan (45 mL)) and stirred overnight to allow evaporation of the organic solvent. The NPs or cNPs were collected and washed with diH_2_O with a Sorvall RC-5B centrifuge, (refrigerated, superspeed). cNPs were pelleted at a speed of 20,000 rpm (47,807× *g*) for 240 min, while NPs were pelleted at a speed of 16,000 rpm (30,590× *g*) for 60 min. Washed NPs or cNPs were dispersed in 2% sucrose in diH_2_O, frozen at −80 °C, freeze-dried (Edwards K4 Modulyo Freeze Dryer, Edwards Vacuum, Crawley, UK), and stored at a −20 °C in a desiccator for the purpose of cryopreservation. Lipophilic tracer DiD with excitation/emission wavelength at 646/663 nm (0.3% *w*/*w*, Invitrogen, Carlsbad, CA, USA) was loaded into NPs or cNPs by addition to the organic phase. Drug-loaded NPs (SL-NPs) or cNPs (SL-cNPs) were made with the addition of simvastatin (4% *w*/*w* drug loading) to the organic phase; empty cNPs (E-cNPs) did not receive drug but did receive DiD. During experiments, low-dose SL-cNPs were defined as using 0.2 mg of SL-cNPs, which contain ~8 µg simvastatin. High-dose SL-cNPs or SL-NPs were defined as using 2 mg of SL-cNPs or SL-NPs, which contain ~80 µg simvastatin.

### 2.3. Nanoparticle Characterization

#### 2.3.1. Hydrodynamic Diameter, Polydispersity Index, and Zeta Potential

Hydrodynamic diameter (size, nm) with particle size distribution and polydispersity index (PDI) were determined with dynamic light scattering, and charge (zeta potential, mV) was calculated after determining electrophoretic mobility, using a Zetasizer Nano ZS90 (Malvern Panalytical, Westborough, MA, USA). cNPs and NPs were suspended in deionized water at a concentration of 0.1 mg/mL and transferred to a disposable polystyrene cuvette or capillary cell. The suspension was equilibrated for 3 min in the cuvette and measured at 90° angle. Each measurement was an average of four separate, consecutive measurements.

#### 2.3.2. Transmission Electron Microscopy

As previously described, for transmission electron microscopy (TEM) imaging, carbon-coated 200 mesh copper grids (Electron Microscopy Sciences, Hatfield, PA, USA), pretreated with 0.002% Alcian blue in 0.03% acetic acid, were floated on top of 30 µL drops of cNP or NP samples for 30 min at room temperature [31]. After washing with diH_2_O, the sample was negatively stained by floating the grid on a 50 µL drop of filtered 2% uranyl acetate (pH 7) for 5 min at room temperature. The sample was allowed to dry for 12 h in a grid storage box, before imaging with a Philips CM120 transmission electron microscope (TSS Microscopy, Beaverton, OR, USA).

#### 2.3.3. Drug Loading

Drug loading was quantified using high performance liquid chromatography (HPLC). Briefly, NPs and cNPs were solubilized in DMSO (4 mg/mL), and compounds were separated on a reversed-phase C18 column with a mobile phase of 70:30 acetonitrile/formic acid (0.05 N). The flow rate was 1 mL per minute. Simvastatin was spectrophotometrically detected by UV absorbance at 240 nm, the retention time was approximately 12 min, and integrated peak areas were extrapolated to an external standard curve.

### 2.4. Cell Culture

ECs (passage 3–10) and VSMCs (passage 3–5) were plated in six-well plates or 25 cm^2^ flasks. Cells were maintained in an incubator at 95% O_2_/5% CO_2_, 37 °C. Cell viability was determined with the Trypan blue exclusion assay using the Countess cell counter (Thermo Fisher Scientific, Waltham, MA, USA) according to the manufacturer’s instructions. Only cells with >90% viability were used.

### 2.5. Flow Cytometry 

ECs or VSMCs were seeded to 50% confluence in 25 cm^2^ flasks. To assess association of NPs or cNPs to ECs or VSMCs, treatment media were prepared by making a stock solution of DiD-encapsulated cNPs or NPs in basal media, as previously described [31]. Cells were treated with either basal media or treatment media (0.2 mg cNPs or NPs) for 30 min. The media were then replaced with fresh basal media and incubated for 24 h. The cells were then harvested and placed into flow cytometry tubes. Greater than 92% viability was confirmed with a trypan blue assay. Flow cytometry was performed by the Loyola University of Chicago Flow Cytometry Core Facility. Acquisition was performed on a four-laser LSRFortessa from BD Biosciences using BD FACSDiva Software (version 9.0). Analysis was performed with FlowJo Software (version 10.9.0) from BD Biosciences (Franklin Lakes, NJ, USA).

### 2.6. Cell Staining

Autoclaved 0.5 mm thickness glass coverslips were placed in six-well plates (Fisher Scientific, Hampton, NJ, USA). ECs and VSMCs were individually seeded to 50% confluence. A stock solution of 2 mg/mL cNPs encapsulated with DiD was made using EC or VSMC basal media. The cells were washed once with PBS and then treated with either basal media or treatment media (0.2 mg cNPs) for 30 min. The media were then removed, cells washed, and fresh basal media were then added to the cells and incubated for 24 h. The cells were fixed and stained with DAPI (4′,6-Diamidino-2-Phenylindole, Dihydrochloride) and Alexa Fluor 488 Phalloidin (Invitrogen, Carlsbad, CA, USA) per the manufacturer’s instructions. Imaging was performed using a fluorescent microscope. The experiment was repeated three times. Uptake of cNPs was determined by visualization of DiD fluorescence (excitation 646 nm, emission 664 nm).

### 2.7. In Vitro Evaluation of SL-cNP Function

ECs and VSMCs were treated with basal media or basal media containing E-cNPs (0.2 mg), free simvastatin (10 µM), low-dose SL-cNPs (0.2 mg), or high-dose SL-cNPs (2 mg). After 30 min, treatment solutions were removed and replaced with fresh basal media. After 24 h, total RNA was extracted from the cells. cDNA was generated and quantitative real-time PCR (qRT-PCR) performed using human-specific primers to *RHOA* or *RHOB* [32]. For each sample, *GAPDH* was used as a reference control.

### 2.8. Rat Carotid Artery Balloon Injury Model

All animal protocols were approved by Loyola University of Chicago IACUC and Edward Hines, Jr Veteran Affairs Hospital IACUC. Age- and weight-matched male Sprague-Dawley rats were purchased at 17–20 weeks at 400 g from Envigo (Indianapolis, IN, USA). Animal age and size were selected for anatomical ease of procedure. In total, six experimental groups (*n* = 6–9 animals for each group) received intraluminal balloon injury using a 2 French Fogarty Catheter (5 atmospheres/5 min, Medline, Northfield, IL, USA) in the common carotid artery. The experimental groups consisted of intraluminal normal saline (control), oral simvastatin plus intraluminal saline, intraluminal cNPs without simvastatin (E-cNPs), intraluminal low-dose SL-cNPs (0.2 mg cNP delivering 8 µg simvastatin), intraluminal high-dose SL-cNPs (2 mg cNP delivering theoretical 80 µg simvastatin), periadventitial high-dose SL-cNPs (2 mg cNP delivering theoretical 80 µg simvastatin), or oral simvastatin (10 mg/kg) plus low-dose intraluminal SL-cNPs. To induce carotid artery balloon injury, the left superior thyroid, occipital, and distal external carotid arteries were ligated. The internal and common carotid arteries were clamped, an arteriotomy was made at the proximal external carotid artery, and a Fogarty Catheter was inserted and inflated at 5 atm for 5 min. After the intraluminal balloon injury was performed, the common carotid artery remained clamped, and the area of injury received an intraluminal bolus of either normal saline (control group), SL-cNPs, or E-cNPs via a 27-gauge blunted needle inserted into the arteriotomy. The bolus (volume 30 µL) was administered gently under pressure and held in place for 30 min, as previously described [33]. After 30 min, the intraluminal therapy was evacuated from the artery. For animals receiving periadventitial SL-cNPs, after balloon injury, the artery was closed, and pluronic gel (100 µg) with high-dose SL-cNP was administered around the common carotid area at the area of injury. Animals receiving oral simvastatin were pretreated with simvastatin (10 mg/kg/day) via chocolate-flavored treats for 1 week prior to surgery and for 14 days postoperatively. Rats receiving oral simvastatin required food rationing to ensure the complete ingestion of the treat. Normal chow was provided ad libitum for the other experimental groups. All rats received water ad libitum. Given IH in this rat model peaks at 14 days, on postoperative day 14, animals were euthanized and perfusion fixed with formalin [33]. Right and left carotid arteries were harvested, preserved in formalin, and sectioned and stained with hematoxylin and eosin. Sections were obtained at 250, 300, 350, and 400 µm from the carotid bifurcation. Morphometric analysis was performed using Motic Images Plus 3.0 (Kowloon Bay, Kowloon, Hong Kong, China) using an intimal/medial area ratio defined as intima/(intima + media). The section with the highest IH ratio was utilized for analysis.

### 2.9. Statistical Analysis

Characterization of nanoparticle size, PDI, and charge was analyzed using Student’s *t*-test, with *p* < 0.05 as significant. RhoA and RhoB mRNA content and intimal hyperplasia were analyzed using one-way and two-way ANOVA, followed by Fishers Least Significant Difference test, with *p* < 0.05 being considered significant. Analysis was performed using GraphPad Prism version 10.0.2 for Windows, GraphPad Software (Boston, MA, USA).

## 3. Results

### 3.1. Characteristics of E-cNPs, SL-cNPs and SL-NPs

**Figure 2:**
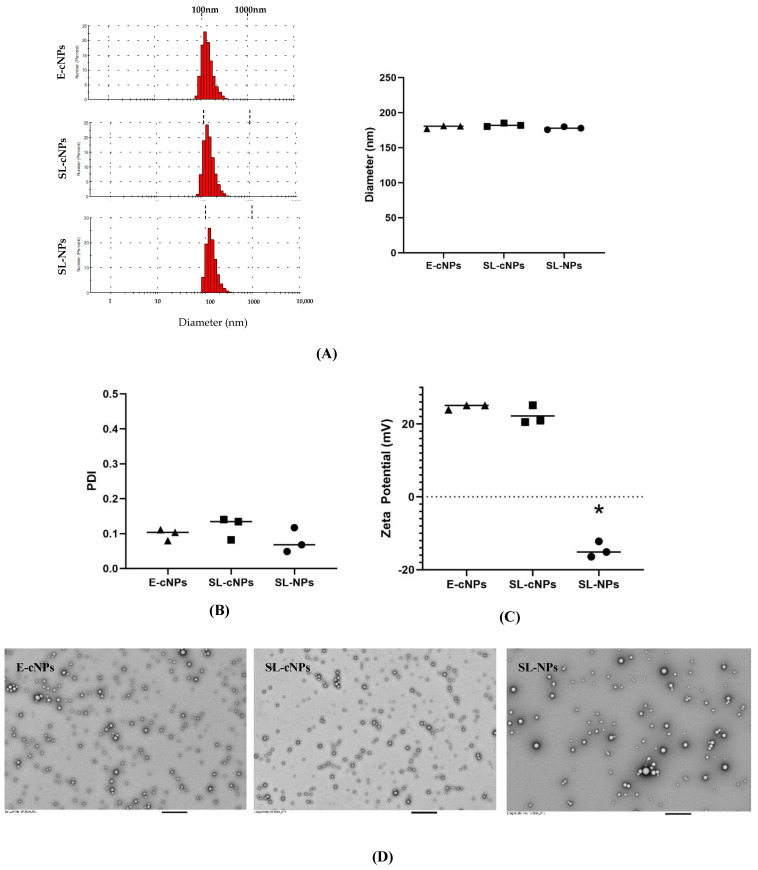
Characterization of SL-cNPs and E-cNPs. Particle size distribution, hydrodynamic diameter (**A**), PDI (**B**), and charge (zeta potential, (**C**)) were determined for E-cNPs, SL-cNPs, and SL-NPs. Data shown as lots around the mean, using 3 separate lots (analyzed using Student’s *T*-test, with * *p* < 0.05 as significant). Transmission electron microscopy was utilized to visualize the E-cNPs, SL-cNPs, and SL-NPs at 5000× magnification; bar represents 800 nm (**D**).

The hydrodynamic diameters of the E-cNPs, SL-cNPs, and SL-NPs were 179.6 ± 1.32 nm, 182.5 ± 1.38 nm, and 177.8 ± 1.24 nm, respectively (Figure 2A). The PDI and zeta potential of the E-cNPs were 0.098 ± 0.009 and +24.69 ± 0.39 mV. PDI and zeta potential of the SL-cNPs were 0.12 ± 0.019 and +22.2 ± 1.47 mV. The PDI and zeta potential of the SL-NPs were 0.08 ± 0.02 and −14.57 mV ± 1.24 (Figure 2B,C). No significant differences existed between the SL-cNPs and E-cNPs. The SL-NPs differed in their zeta potential, compared to the cNP formulations due to the presence of chitosan resulting in a positively charged surface (Figure 2C). TEM was then utilized to visually appreciate the E-cNPs, SL-cNPs, and SL-NPs (Figure 2D). The actual drug loading of simvastatin in the cNPs was 2.77% *w*/*w*, resulting in a calculated encapsulation efficiency (actual drug loading/theoretical drug loading × 100%) of 71.78 ± 2.86%. We utilized 0.2 mg of SL-cNPs as low-dose (effectively locally administering 5.5 µg simvastatin) and 2 mg SL-cNPs as high-dose (effectively locally administering 55 µg simvastatin). Cumulative drug release was determined previously and approached 100% at 100 h [31].

### 3.2. SL-cNPs Readily Associate with ECs and VSMCs Compared to SL-NPs

**Figure 3:**
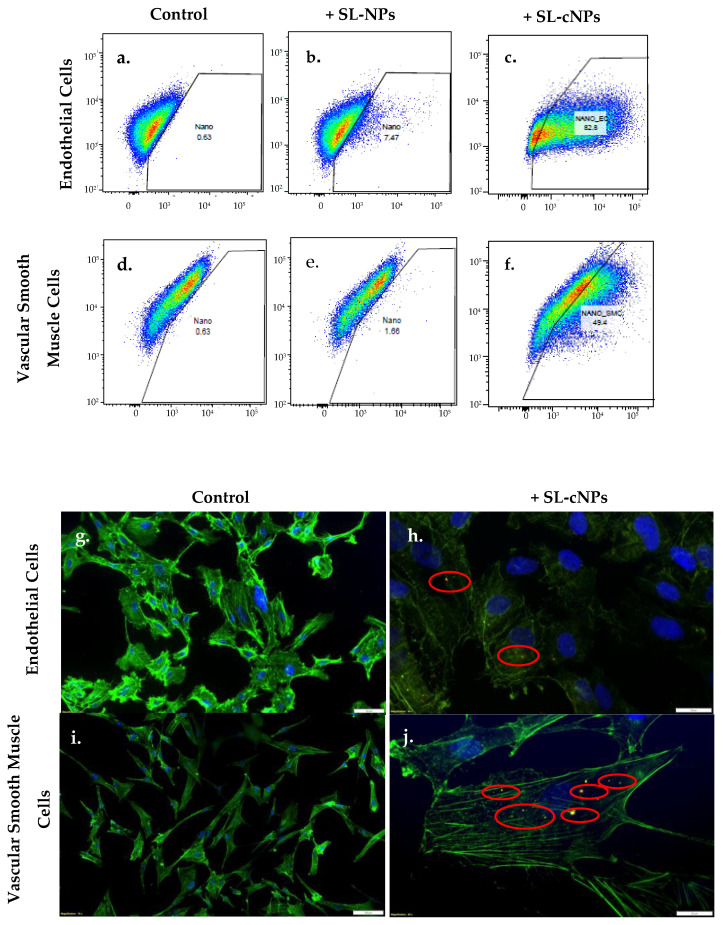
SL-cNPs encapsulated with DiD lipophilic tracer readily associate with ECs and VSMCs. Human ECs (**a**–**c**) or VSMCs (**d**–**f**) were cultured with either basal media or basal media with 0.2 mg SL-cNPs or with 0.2 mg SL-NPs for 30 min. For flow cytometry (**a**–**f**), cells were harvested and dispersed into flow cytometry tubes with basal media. SL-cNP- and SL-NP-positive cells elicited an emission wavelength at 664 nm. ECs and VSMCs treated with SL-cNPs demonstrated increased association to SL-cNPs as compared to control cells and cells treated with SL-NPs. Immunostaining was then preformed to visualize association of SL-cNPs to cells (**g**–**j**). Cells were fixed and labeled with DAPI (blue) or Alexa Fluor 488 Phalloidin (green). Red fluorescence signifies DiD-encapsulated SL-cNPs (outlined with red circle).

ECs and VSMCs exposed to SL-cNPs demonstrated increased allophycocyanin (APC) intensity in flow cytometry (Figure 3a–f) compared to the non-treated and SL-NP-treated cells. Analysis demonstrated association (positive APC fluorescence) to ECs following treatment with SL-cNPs was 79.6 ± 1.6%, while treatment with SL-NPs was 6.5 ± 0.6% (*p* < 0.05). Association to VSMCs after treatment with SL-cNPs was 46.4 ± 1.8, while treatment with SL-NPs was 1.6 ± 0.07% (*p* < 0.05), thereby demonstrating the positive charge elicited by chitosan improves adhesion to cells. Representative associations of SL-cNPs are visualized using immunostaining (Figure 3h,j). We proceeded with our experiments using SL-cNPs and not SL-NPs given the stark differences in cellular association. 

### 3.3. SL-cNPs Increase RhoA and RhoB mRNA Content in ECs and VSMCs

To determine whether SL-cNPs have a functional effect on ECs and VSMCs, we assessed the gene expression of RhoA and RhoB after treatment. Statins inhibit HMG-CoA reductase, isoprenoid production, and GTPase activity, resulting in a compensatory increase in RhoA and RhoB mRNA content [32]. Therefore, we expect the administration of statins to increase RhoA and RhoB gene expression.

**Figure 4:**
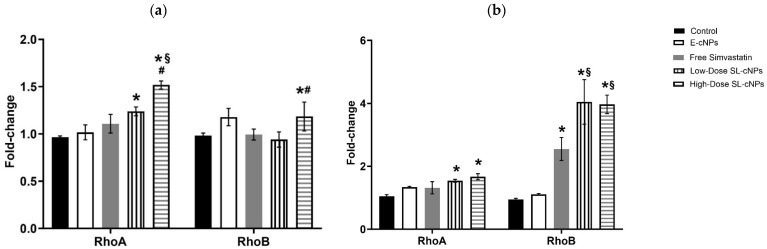
SL-cNPs enhance RhoA and RhoB mRNA content in EC and VSMC cultures. (**a**) Endothelial cells; (**b**) vascular smooth muscle cells. Confluent ECs or VSMCs were cultured in basal media without or with E-cNPs, free simvastatin, or low-dose or high-dose SL-cNPs for 30 min and then washed. Relative changes in RhoA and RhoB mRNA content were quantified by RT-qPCR. Data shown are means ± SEM (*n* = 3) and are expressed as GAPDH-normalized fold-changes. * *p* < 0.05 compared to control; **#** *p* < 0.05 compared to low-dose SL-cNPs; § *p* < 0.05 compared to free simvastatin; statistical analysis performed using ANOVA.

ECs and VSMCs cultured in the presence of E-cNPs demonstrated no significant difference in expression of RhoA or RhoB compared to control cells (Figure 4a,b). ECs cultured in the presence of low-dose SL-cNPs had a 1.28-fold increase in RhoA mRNA expression while high-dose SL-cNPs demonstrated a 1.57-fold increase compared to control ECs (*p* < 0.05, Figure 4a). High-dose SL-cNPs induced increased RhoA mRNA expression compared to free simvastatin and low-dose SL-cNPs (a 1.34- and 1.27-fold increase, respectively, *p* < 0.05). Only high-dose SL-cNP demonstrated increased RhoB expression, with a 1.20- and 1.26-fold increase in RhoB mRNA compared to control and low-dose SL-cNP, respectively (*p* < 0.05). Low-dose SL-cNP and free simvastatin expression of RhoB was not significantly different compared to control.

RhoA mRNA expression was significantly increased in VSMCS cultured in the presence of low- or high-dose SL-cNPs (a 1.42- and 1.61-fold increase, respectively, Figure 4b). RhoB expression was significantly increased after VSMC treatment with free simvastatin, low-dose and high-dose SL-cNPs (2.68-, 4.26-, and 4.17-fold increases, respectively). Low-dose and high-dose SL-cNPs demonstrated increased expression of RhoB compared to VSMCs treated with free simvastatin (1.59- and 1.55-fold increases, respectively).

### 3.4. Oral Simvastatin and SL-cNPs Reduce IH Following Carotid Artery Balloon Injury

**Figure 5:**
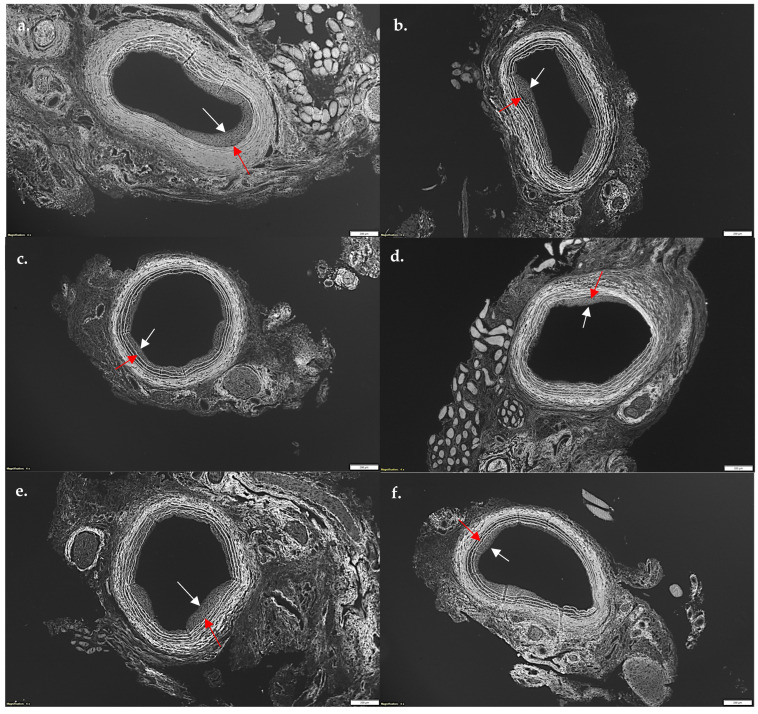
Photomicrograph of representative cuts of intimal hyperplasia after balloon injury of carotid arteries. Rats were sacrificed 14 days after balloon injury, and carotid arteries were harvested, perfused, fixed, sectioned, and stained with hematoxylin and eosin. Utilizing autofluorescence, intimal hyperplasia was measured using intimal/medial area ratios. (**a**) No statin control; (**b**) empty-cNPs; (**c**) oral simvastatin; (**d**) periadventitial SL-cNPs; (**e**) intraluminal SL-cNPs; (**f**) intraluminal SL-cNPs and oral simvastatin. Arrows indicate areas of intimal hyperplasia. White bars represent 200 µm.

**Figure 6:**
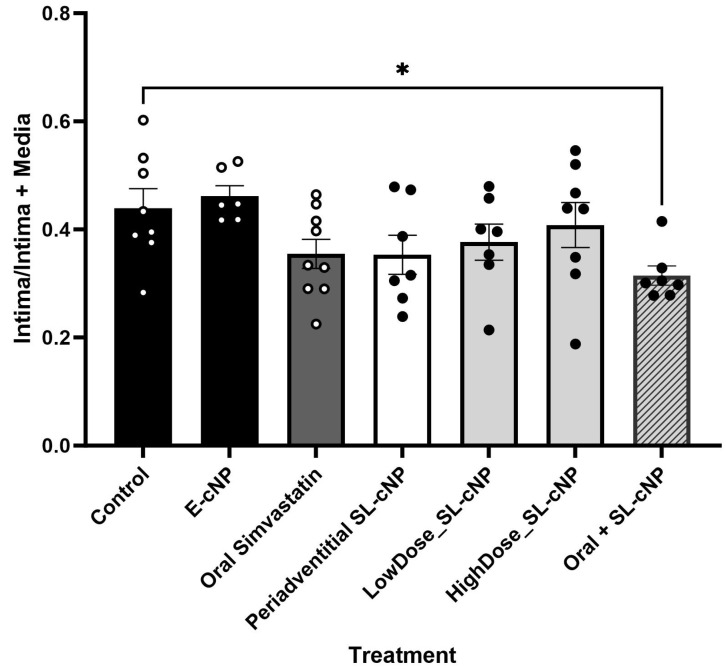
Intima/intima + media ratios. Oral simvastatin in combination with intraluminal administration of low-dose SL-cNP significantly reduced intimal hyperplasia compared to control rats. Data presented as means ± SD. Control (*n* = 8), E-cNP (*n* = 7); oral simvastatin (*n* = 9); periadventitial SL-cNPs (*n* = 7),; low-dose SL-cNP (*n* = 7); high-dose SL-cNPs (*n* = 8); oral + SL-cNP (*n* = 7). Analysis was performed using one-way ANOVA; *, *p* < 0.05.

Given SL-cNPs demonstrated increased association to ECs and VSMCs in vitro and demonstrated an effect on gene expression, we assessed their effect on IH in vivo. Localized treatment of either high- or low-dose SL-cNPs was assessed by either administering the cNPs intraluminally as a bolus held in place for 30 min, then removed, or administered periadventitially. Periadventitial administration was injected surrounding the carotid artery following closure of arteriotomy. Mean ± SEM IH ratios were as follows: control animals 0.440 ± 0.04, intraluminal empty-cNPs 0.416 ± 0.05, oral simvastatin 0.35 ± 0.03, high-dose periadventitial SL-cNP 0.35 ± 0.04, low-dose intraluminal SL-cNP 0.377 ± 0.03, high-dose intraluminal 0.41 ± 0.04, and oral + SL-cNPs 0.34 ± 0.03. Representative images of IH are presented in Figure 5a–f (red and white arrows demonstrating area of IH). Only oral simvastatin with the addition of low-dose intraluminal SL-cNPs significantly reduced IH by 23% (0.34 ± 0.03, *p* < 0.05) compared to the untreated control group. The data are summarized in Figure 6.

## 4. Discussion

Restenosis secondary to IH after PTA for PAD remains an ongoing challenge that decreases long-term patency rates and limb preservation [1,2,3]. Although the clinical use of FDA-approved paclitaxel-coated balloons and stents has represented a major advance in the prevention of IH, particularly in coronary artery disease, there remains controversy regarding their benefit in PAD lesions below the knee [9,10,11,12]. Therefore, the purpose of this study was to examine the feasibility of a unique localized treatment, SL-cNPs, to reduce IH after PTA. We found that SL-cNPs (1) readily associate with ECs and VSMCs in vitro; (2) affect VSMC and EC function, as demonstrated by compensatory increases in gene expression in vitro; and (3) reduce IH when used in combination with oral simvastatin in vivo. To our knowledge, the use of statin-loaded, chitosan-functionalized nanoformulations to limit IH formation after balloon injury is novel.

Clinical studies since the late 1990s have demonstrated that statins have beneficial pleiotropic effects independent of lowering cholesterol levels. These studies have demonstrated that statins reduce major adverse cardiovascular events, have anti-inflammatory properties, improve PAD symptoms, and increase circulating endothelial progenitor cells [17]. At the cellular level, statins interfere with IH by decreasing the local inflammatory response (i.e., reducing leukocyte EC transmigration), inhibiting VSMC migration from the adventitial layer to intimal layer, and improving reendothelialization by increasing endothelial progenitor cell survival [8,17,34]. Nevertheless, systemic delivery of statins has not proven to be highly effective in preventing IH due to statin’s low solubility, rapid metabolism, and low bioavailability [25]. More recently, novel drug delivery mechanisms, such as microparticles, micelles, and NPs, have been developed to prevent IH [24,25,28,29,35]. Of these carriers, FDA-approved polymeric NPs, such as PLGA-NPs, are being considered the new frontier of medicine, given their ability to provide precise targeting, sustained drug release, and improved bioavailability [25,26,31,32,36]. Furthermore, by modifying the surface composition of the nanomaterial, NPs provide new strategies for passive or active targeting [25]. The variations in NP formulation raises questions concerning the optimal drug delivery vehicle(s) for IH prevention. Of the various formulations of PLGA-NPs, we chose to utilize chitosan-functionalized PLGA-NPs due to chitosan’s inherent beneficial properties. First, chitosan is polycationic, which increases the bioadhesion and solubility of NPs compared to PLGA alone [30,31]. Second, chitosan increases the stability, bioavailability, and controlled release of the encapsulated drug. Third, chitosan contains inherent antimicrobial and anti-inflammatory properties [30,37]. cNPs have also been shown to have satisfactory biocompatibility with normal developing cells and have been safely utilized in oral, eye, cutaneous, and transdermal applications [30,31]. Furthermore, chitosan has also been used in the surgical field with applications in surgical sutures, wound dressings, defect fillers, and tissue-engineering scaffolds, demonstrating its wide range/ease of use [38]. Regarding the use of simvastatin within our cNP formulation, previous studies have demonstrated systemic and local delivery of simvastatin reduces IH [24]. We elected to utilize low- (0.2 mg) and high-dose (2 mg) SL-cNPs as this would provide local delivery of 5.5 μg or 55 μg simvastatin, respectively. We elected to assess varying doses of simvastatin that were significantly reduced compared to systemic therapy (10 mg/kg/day) to minimize any potential negative off-target side effects while maximizing therapeutic efficacy [17,39]. Furthermore, we proceeded with the low-dose SL-cNP as it is similar to the dose we have used in a previous study [24].

Our in vitro results demonstrate that chitosan-functionalized nanoparticles associate more strongly with ECs and VSMCs than PLGA nanoparticles alone, and this is likely a result of chitosan imparting a positive surface charge. This prompted us to assess SL-cNPs’ cellular effect on ECs and VSMCs by quantifying the relative gene expression of RhoA and RhoB in vitro. We elected to assess gene expression as previous studies have demonstrated statin’s inhibition of isoprenoid intermediates leads to a compensatory increase in constitutive RhoA and inducible RhoB mRNA expression [32,40]. RhoA expression is particularly relevant to our study as statins’ inhibition of RhoA is a suspected pleiotropic mechanism that leads to IH reduction. For example, RhoA, a small GTP binding protein, interacts with Rho-kinase to promote vascular contraction and VSMC migration by phosphorylating myosin light chain and myosin phosphatase, target subunit 1 [41]. RhoA/Rho-kinase is also implicated in reducing nitric oxide synthase gene expression, an important mediator of EC and VSMC function [42]. RhoA expression also positively modulates the expression of endothelial adhesion molecules, such as P-and E-selectins, intercellular adhesion molecule (ICAM)-1, and vascular cell adhesion molecule (VCAM)-1, that are important for leukocyte–endothelial adhesion and transmigration [21,22]. Therefore, inhibition of RhoA is suspected to reduce IH by inhibiting VSMC migration and the activation of a vascular inflammatory milieu. In our study, we found low- and high-dose SL-cNPs significantly increased the expression of RhoA in ECs and VSMCs. Only high-dose SL-cNPs increased RhoB expression in ECs, while RhoB expression was increased in VSMCs treated with low- or high-dose SL-cNPs. Furthermore, SL-cNPs demonstrated a more robust effect on RhoA and RhoB mRNA expression compared to free simvastatin alone. We suspect free simvastatin did not have a robust effect as treatment was for 30 min. The adhesion properties of the SL-cNPs are therefore exposing the cells to a longer duration of simvastatin than free simvastatin alone. Our results suggest that SL-cNPs have the potential to reduce IH through its effect on RhoA and, given the more robust effect on gene expression compared to free simvastatin, may have greater therapeutic efficacy in the acute period (30 min).

Although our in vivo results did not demonstrate a significant effect of oral simvastatin, periadventitial SL-cNPs, low-, or high-dose intraluminal SL-cNPs alone, the combination of low-dose SL-cNPs and oral simvastatin was effective in reducing IH. This may be a result of oral simvastatin providing a baseline circulatory inhibition on isoprenoid intermediates, while the SL-cNPs provide a localized acute effect that facilitates simvastatin efficacy in IH prevention. Therefore, the compound effect of SL-cNPs and oral simvastatin suggests further investigation is necessary to assess the optimal dose, timing, and/or route of administration that would enhance SL-cNPs’ inhibitory effect on IH. For example, a previous study using a porcine coronary model demonstrated a pitavastatin-loaded NP eluting stent significantly reduced IH when 20 μg of pitavastatin was utilized per stent [29]. In our study, we examined the use of intraluminal administration of 5.5 μg or 55 μg of simvastatin loaded in cNPs for 30 min before restoring blood flow, with subsequent removal or periadventitial administration (55 µg). Although a 30-min duration appears to be a feasible mechanism to administer SL-cNPs, we expect a different route of administration with prolonged exposure to the intima may demonstrate a more profound effect on IH; however, the prolonged exposure of SL-cNPs periadventitialy did not demonstrate a robust effect either. When assessing the systemic effect of simvastatin, in a previous study, we found a significant reduction of IH by 25% when oral simvastatin was used in isolation [24]. In this study, oral simvastatin did not have a statistically significant impact, although there was a 20% reduction in IH. We expect the effect of simvastatin may vary among animals, and, in order to maximize statin’s pleiotropic benefits, systemic simvastatin may need to be used in combination with localized therapy to provide not only circulatory isoprenoid inhibition but localized inhibition in the acute phase of injury as well [43].

The current study is primarily one of feasibility, and it does have limitations. One limitation includes the delivery mechanism of SL-cNPs. Our results cannot be compared to previous statin–NP formulations as we assessed a temporary intraluminal treatment, while previous studies have utilized NP-coated permanent stents. We elected to test our hypothesis with a short-term treatment approach as, clinically, this treatment would be ideal after balloon angioplasty. Another limitation includes the lack of documented localization of SL-cNPs to the arterial wall. Future directions include optimizing the delivery of cNPs, which can be accomplished by using different nanoformulation sizes and concentrations of SL-cNPs and examining different delivery routes of SL-cNPs administration. Given the release kinetics of SL-cNPs, we expect statins have a profound effect in the acute period, resulting in decreased IH.

## 5. Conclusions

In conclusion, for IH prevention, chitosan-functionalized nanoformulations are feasible but may not be the best vehicle to locally deliver statins to vascular cells. Future studies should evaluate the optimal size, concentration, delivery, and residence time of SL-cNPs to maximize statins’ effect or look at different NP formulations to optimize IH prevention.

## Data Availability

The datasets generated during and/or analyzed during the current study are available from the corresponding author on reasonable request.

## Abbreviations

The following abbreviations are used in this manuscript:

ECs	Endothelial cells
VSMCs	Vascular Smooth Muscle Cells
IH	Intimal Hyperplasia
PAD	Peripheral arterial disease
PTA	Percutaneous transluminal balloon angioplasty
PLGA	Poly-lactic-co-glycolic acid
cNPs	Chitosan-functionalized PLGA nanoparticles
SL-NPs	Simvastatin-loaded PLGA nanoparticles
SL-cNPs	Simvastatin-loaded chitosan-functionalized PLGA nanoparticles
E-cNPs	Empty chitosan-PLGA nanoparticles
